# Subinhibitory Concentrations of Prim-O-Glucosylcimifugin Decrease the Expression of Alpha-Hemolysin in* Staphylococcus aureus* (USA300)

**DOI:** 10.1155/2018/7579808

**Published:** 2018-07-19

**Authors:** Ouyang Ping, Yang Ruixue, Deng Jiaqiang, Wang Kaiyu, Fang Jing, Geng Yi, Huang Xiaoli, Chen Defang, Lai Weimin, Tang Li, Yin Lizi

**Affiliations:** ^1^College of Veterinary Medicine, Sichuan Agriculture University, Chengdu, China; ^2^College of Animal Science and Technology, Sichuan Agriculture University, Chengdu, China

## Abstract

*Staphylococcus aureus (S. aureus)*, an important opportunistic pathogen in human and animal, causes a series of diseases in the impairing of immunity of host and even then death. Alpha-hemolysin (Hla), a primary virulence factor, plays a major role in the pathogenic progress of* S. aureus*, especially in pneumonia. Prim-O-glucosylcimifugin (POG), a nature chromone compound, is an active ingredient in many Chinese Medicines. In this study, POG investigated the inhibitory effect of the secretion of Hla in* S. aureus* strain USA300 at the subinhibitory concentrations. The hemolysis assays and western blotting assays showed that POG can decrease the production of Hla in the USA300 growth cell cultures in a dose-dependent manner. The results of RT-PCR revealed that reduction of Hla was related to inhibit the transcription of* hla* and* RNAIII*. In the cells experiment, POG was proved to protect A549 cells from Hla-medicated injury. In conclusion, POG was shown the capacity of decreased the production of* S. aureus* Hla. POG can be developed as a candidate agent to treat* S. aureus* infections against Hla.

## 1. Introduction

*Staphylococcus aureus (S. aureus*), a common Gram-positive bacterium pathogen in clinic, can cause various infectious diseases in skin, respiratory system, and bloodstream in human and animals [1].* S. aureus *infection is an important zoonosis which impacts both human and animals. USA300 strain, a strain of community-associated methicillin-resistant* S. aureus *(MRSA), was first identified in 1998 in USA [2]. USA300 strain is causing diseases in many countries and regions around the world over the last decade [3–5].* S. aureus* USA300 has become one of the most globally spread MRSA strain and caused a worldwide epidemic such as skin infection, soft tissue infections, and severe pneumonia [4]. In general, conventional antimicrobials are used to treat the bacterial diseases by killing the bacteria or inhibiting bacteria generation. Antibiotics increase selective pressure in sensitive bacteria, screen out resistant strains, and accelerate the spreading on global [6]. With widespread application and abusing of antibiotics, bacterial resistances are becoming a growing problem. With the widespread MRSA epidemics, MRSA is listed as a “serious threat” in CDC (Center for Disease Control and Prevention) [7]. Consequently, novel agents and therapeutic strategies are urgently needed to treat bacterial infections, especially of antibiotic-resistant bacteria.

It was proved that the pathogenicity of* S. aureus* is related to the virulence factors of* S. aureus *[1]. Montgomery et al. (2008) have proved that USA300 isolates had stronger pathogenicity and caused more severe pneumonia in rat pneumonia model than USA400 isolates [8]. Virulence factors, like Panton-Valentine leukocidin and alpha-hemolysin (Hla), were increasingly secreted in USA300 isolates [8, 9]. Hla is an important virulence protein that is secreted by* S. aureus* into the extracellular. Hla causes disease by damaging various cells and tissues [10, 11]. Pneumocytes are effective target cells of Hla [11, 12]. Many researches have proved that Hla plays an important role in the pathogenesis of* S. aureus* infections, especially in pneumonia [10, 13, 14]. Consequently, Hla is considered a candidate drug target for the treatment of MRSA infections such as deadly staphylococcal pneumonia [15]. Due to the indispensable character of Hla in the pathogenicity of* S. aureus*, drugs reacting on the Hla will be novel medicines in* S. aureus* infection.

**Figure 1:**
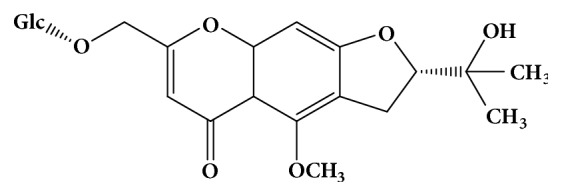
The chemical structure of prim-O-glucosylcimifugin (CAS No: 80681-45-4).

As a familiar chromone, prim-O-glucosylcimifugin (POG, chemical structure shown in Figure 1) is one of* Saposhnikovia divaricata* (Turcz) Schischk's major effective components. In Chinese Pharmacopoeia, POG has been defined as index component and active ingredient standardized of* Saposhnikovia divaricata* (Turcz) Schischk [16, 17]. Previous researches showed that POG exhibited many potent pharmacological activities including anticancer, analgesic, anticonvulsant, antipyretic, antinociceptive, and anti-inflammatory effects [18–21].* Saposhnikovia divaricata* (Turcz) Schischk was commonly used for treatment of common colds and headache in traditional Chinese medicine. To our knowledge, no study had shown the effects of POG on the expression of Hla in* S. aureus*. In this study, we evaluated the effect of POG on the inhibition of Hla secretion in* S. aureus* USA300 using the hemolysis assay, western blotting, RT-PCR, and cell experiments.

## 2. Materials and Methods

### 2.1. Bacterial Strains, Cell Line, and Reagents

The community-associated methicillin-resistant* S. aureus* (CA-MRSA) strain USA300 (ATCC BAA-1717), a Hla-producing strain, was used in this study. This strain was purchased from the American Type Culture Collection (ATCC). In this cells experiment, A549 cells (human alveolar epithelial cell line, ATCC CCL185) were used and commercially obtained from ATCC. Prim-O-Glucosylcimifugin (POG, purity≥98%) was obtained from Chengdu Herbpurify Co., Ltd., (Chengdu, China). The solution (40960 *μ*g/mL) of POG was prepared by dimethyl sulfoxide (DMSO; Sigma-Aldrich) and stored in 4°C for* in vitro* study.

### 2.2. Susceptibility Testing

The minimal inhibitory concentrations (MICs) of POG against* S. aureus* strain were determined by the broth microdilution method which recommended by the Clinical and Laboratory Standards Institute. Shortly, POG was diluted to the concentration range of 2-512 *μ*g/mL in a 96-well plate by double dilution method. Bacteria (5 × 10^5^ CFU/mL) were inoculated to each well. The plate was inoculated at 37°C for 24 h. The MIC was defined as the lowest drug concentration that inhibited bacteria growth and was repeated for three times.

### 2.3. Growth Curve Assay

Bacteria were cultured in TSB at 37°C to OD_600_ = 0.3 and equally (100 ml) divided into 7 flasks (250 mL), followed by the accretion of POG at concentrations of 0, 4, 8, 16, 32, 64, and 128 *μ*g/mL. These flasks were placed in an incubator with 37°C and constant shaking (200 rpm) under aerobic conditions. Bacteria growth was determined by reading the OD_600_ values of the cultures every 30 min.

### 2.4. Hemolysis Assay

Bacteria were grown in TSB at 37°C, with different concentrations of POG. When bacteria reached the postexponential growth phase (OD_600_ of 2.5), cell culture was centrifuged (1000×g, room temperature, 2 min). The supernatants were collected and stored in sterile tube. Twenty-five *μ*L defibrinated rabbit red cells and 100 *μ*L of supernatant were added to 875 *μ*L sterile PBS. The mixtures were tenderly mixed well and incubated at 37°C chamber for 15 min, and then the mixtures were centrifuged by 10,000×g at 20°C for 1 min. The OD_543_ values of the supernatant of mixtures were measured by spectrophotometer (Agilent Technologies). The hemolytic activities were reflected by the OD_543_ values. Negative control (without POG) was served as 100% hemolysis. The hemolysis percentage of drug group was calculated by comparison with drug-free control.

### 2.5. Western Blotting

The bacteria culture supernatants, which were collected from the hemolysis assay, were used for western blotting analysis. The protocol of the western blotting was performed as described previously [22]. The proteins in the supernatants were separately denatured via boiling with Laemmli SDS buffer for 5 min. Subsequently, target protein was separated by sodium dodecyl sulfate (SDS) and polyacrylamide (12%) gel. Hla was detected using ECL western blotting detection reagents (Bio-Rad, ChemiDoc™ MP). The antibody against* S. aureus* Hla and the secondary antibodies (an anti-rabbit antiserum conjugated with horseradish peroxidase) were obtained from Sigma-Aldrich.

### 2.6. RNA Isolation and RT-PCR Assay

Bacteria cultures were the same of the hemolytic activity assay. Bacteria were centrifuged by 5000×g at 4°C for 5 min. The cell pellets were resuspended and lysed in TES buffer. Total RNAs of samples were extracted by using the RNeasy mini kit (Qiagen) according to the manufacturer's instructions. DNA was removed from each RNA preparation using RNase-free DNase I (Qiagen) according to the manufacturers' directions for rigorous DNase treatment. The OD_260_ of the purified RNA was measured by a UV spectrophotometer (Agilent Technologies). cDNA was synthetized from extracted RNA using iScript cDNA synthesis Kit (Bio-Rad). PCR amplification was assessed by Real-Time System (CFX ConnectTM, Bio-Rad Laboratories, Inc., USA). All samples were analyzed in triplicate. The 16S rRNA was used (housekeeping gene) as a reference gene. In this study, the relative expression of a target gene versus the 16S rRNA gene was utilized to determine the changes in the transcript-level between samples. The results were analyzed with ABI Prism 7000 SDS software. PCRs were performed with the primers that are listed in Table 1.

### 2.7. Viability and Cytotoxicity Assay

A549 cells (ATCC CCL 185) were cultured in Dulbecco's Modified Eagle's Medium (DMEM) contained heat-inactivated fetal calf serum (10%, Bioind) and penicillin/streptomycin (100 U/ml, Sigma). Cells were seeded on 96-well culture plates at a cells concentration of 2.0 × 10^5^ cells each well for 18 h at 37°C incubator with 5% CO_2_.* S. aureus *USA300 was multiplied in TSB at 37°C. When the OD_600_ of bacterial cultures were 0.5, bacteria were collected from 5 ml culture medium by centrifuge (1 min, 1000×g, 4°C). The pellets were washed with sterile PBS three times. And then, the bacteria were resuspended in 10 ml of DMEM (without penicillin/streptomycin) to get bacterial suspension. One hundred *μ*L bacterial suspensions were added to each well seeded with A549 cells in 96-cell dishes. A549 cells with bacteria were cultured 6 h at 37°C with indicated concentrations of POG. The morphology and growth condition of A549 were determined via the live/dead (green/red) reagent (Invitrogen) and by Cytotoxicity Detection kit (LDH) (Roche, Switzerland), respectively. The microscopic images of stained cells were obtained using a confocal laser scanning microscope (Nikon, Japan). The amounts of released LDH in mix-culture were measured by a microplate reader (Tecan, Austria) at an absorbance of 490 nm. All experiments were accorded manufacturers' directions.

### 2.8. Statistical Analysis

The results were analyzed for significance using an independent Student's* t*-test, and a P value of < 0.05 was considered to be statistically significant. The statistical analyses were performed using the SPSS 13.0 statistical software.

## 3. Results

### 3.1. Effect of Prim-O-Glucosylcimifugin on* S. aureus* Growth

**Figure 2:**
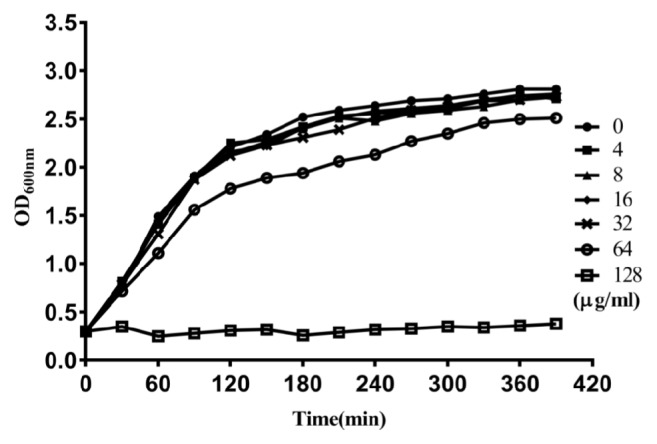
Growth curves of* S. aureus* USA300 strain in TSB with or without POG. Symbols ●, ■, ▲, ◆, x, ○, and □ represent* S. aureus* USA300 strain grown in TSB with 0, 4, 8, 16, 32, 64, and 128 *μ*g/mL of POG, respectively.

The minimal inhibitory concentration (MIC) of POG against* S. aureus* USA300 was 128 *μ*g/mL. Additionally, we detected the growth curve of* S. aureus* USA300 treated with different concentrations of POG (4 to 32 *μ*g/mL). The growth curves showed that POG did not inhibit the multiplication of bacterium in POG-treated groups and negative group (Figure 2). These data showed that POG had a little anti-*S. aureus* activity, but there were no influence on the multiplication of* S. aureus* in the range of experimental concentration.

### 3.2. Prim-O-Glucosylcimifugin Inhibits the Hemolytic Activity of Coculture Supernatant

**Figure 3:**
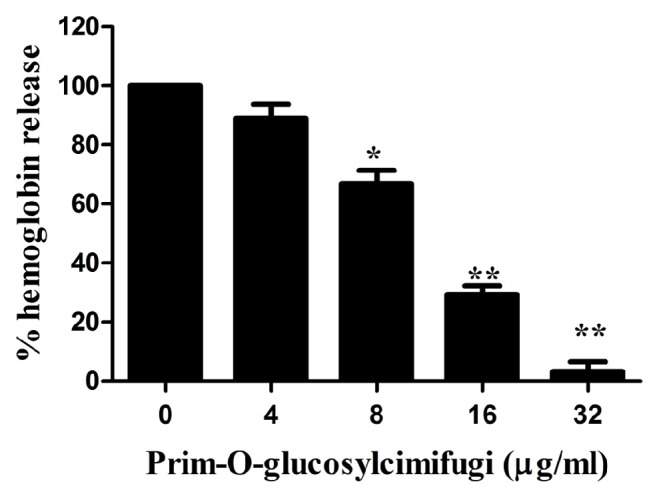
Hemolytic activity of Hla produced by* S. aureus *USA300 coculture with subinhibitory concentrations of prim-O-glucosylcimifugin. The data shown are representative of three independent experiments. *∗* indicates P < 0.05 and *∗∗* indicates P < 0.01, compared with the prim-O-glucosylcimifugin-free culture.

Hemolytic activities of bacteria supernatants cocultured with or without POG were investigated using the hemolysis of rabbit blood red cells.  Figure 3 showed that POG inhibited the hemolytic activity in a dose-response relationship (from 4 to 32 *μ*g/mL) in coculture supernatants. When USA300 was cultured with 16 *μ*g/mL of POG, the hemolysis value of culture supernatant was 29.3% (P < 0.01) compared with the negative control. Almost no hemolysis (3.2%) was detected at the concentration of 32 *μ*g/mL. Additionally, POG did not lyse rabbit blood red cells and had no effect on the hemolysis induced by pure Hla (Sigma-Aldrich) with the concentration up to 64 *μ*g/mL (date not shown). The hemolytic activity was inhibited by POG in USA300 culture supernatant with a dose-response manner in this study.

### 3.3. Prim-O-Glucosylcimifugin Decreases the Secretion of Hla in Culture Supernatant

**Figure 4:**
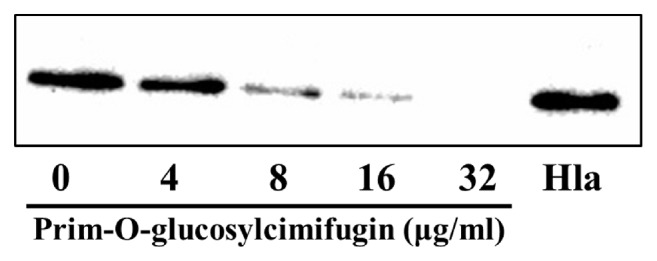
The production of *α*-hemolysin (Hla) in the culture supernatants of* S. aureus* USA300 grown with or without prim-O-glucosylcimifugin.

The relation between decreased hemolysis and the content of Hla in culture medium was confirmed via western blotting assay. In accordance with the hemolysis assay, POG decreased the secretion of Hla in a concentration-dependent manner (Figure 4). A recognizable reduction in Hla secretion was observed in the group that treated with 16 *μ*g/mL of POG. Nevertheless, almost no target protein was observed in* S. aureus* USA300 treated with 32 *μ*g/mL POG.

### 3.4. Prim-O-Glucosylcimifugin Attenuates* hla* and* RNAIII* Transcription in* S. aureus*

**Figure 5:**
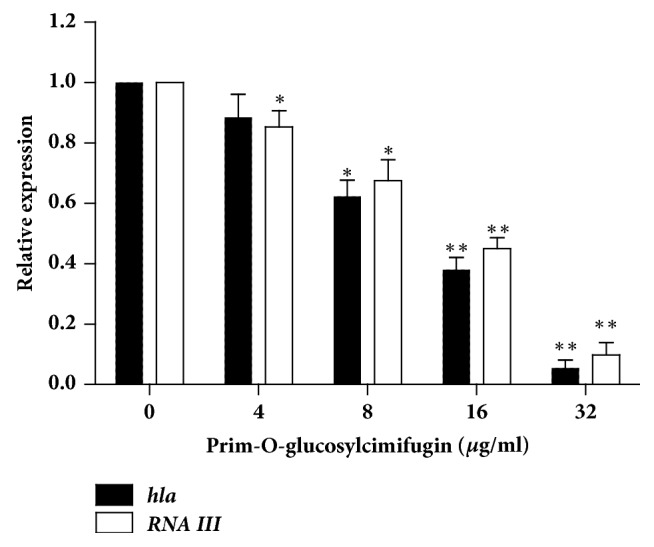
The relative expression of* hla* and* RNAIII *in* S. aureus* USA300 after growing in the absence or in the presence prim-O-glucosylcimifugin. The data shown are representative of three independent experiments. *∗* indicates P < 0.05 and *∗∗* indicates P < 0.01, compared with the prim-O-glucosylcimifugin-free culture.

Alpha-hemolysin is coded by the* hla* gene. The real-time RT-PCR was used to detect the transcriptions of* hla* in* S. aureus* USA300 treated with POG doses ranging from 4 to 32 *μ*g/mL. The* arg* regulatory system is a positive regulator of Hla in* S. aureus*. In* S*.* aureus*, the products of more than 100 virulence factors (including Hla) were positively associated with RNAIII, an effector molecule of the* agr* system [23, 24]. The transcription of* RNAIII* was also investigated. Our results showed that the transcriptions of* hla* and* RNAIII* were reduced in* S. aureus* USA300 treated with POG by a dose-dependent relationship (Figure 5). When USA300 was cultured with POG in the concentration of 32 *μ*g/mL, the relative transcription levels of* hla* and* RNAIII* in USA300 were observably decreased (5.5% and 9.8% for* hla* and* RNAIII*, respectively) compared with negative control group.

### 3.5. Prim-O-Glucosylcimifugin Alleviates Hla-Mediated A549 Cells Injury

**Figure 6:**
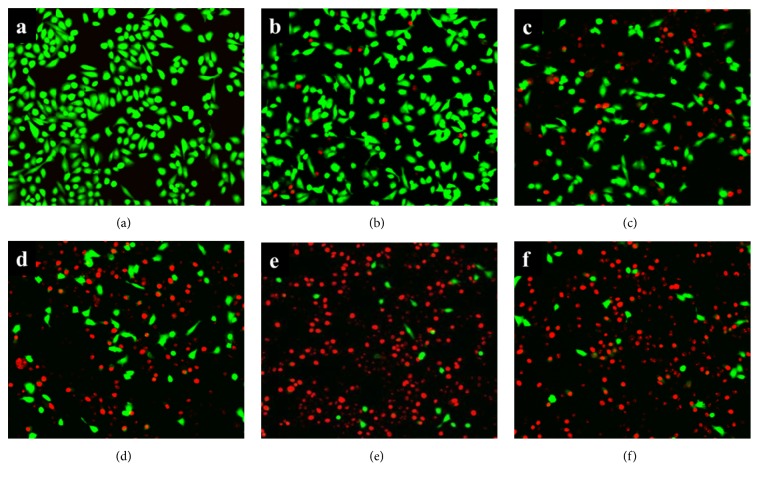
The situation of live and dead cells in the A549 human alveolar epithelial cell coculture with* S. aureus* USA300 treated with different concentrations of prim-O-glucosylcimifugin. Live/dead reagent-stained A549 was observed with fluorescent imaging (100×), using calcein AM and ethidium homodimer-1 (EthD-1), respectively. (a) Uninfected A549 cells; (b) A549 cells was infected by* S. aureus *USA300 with 32 *μ*g/ml of POG; (c) A549 cells was infected by* S. aureus* USA300 with 16 *μ*g/ml of POG; (d) A549 cells was infected by* S. aureus* USA300 with 8 *μ*g/ml of POG; (e) A549 cells was infected by* S. aureus* USA300 with 4 *μ*g/ml of POG; (f) A549 cells was infected by* S. aureus* USA300 without POG.

Human alveolar epithelial A549 cell is usually used as cell model for pulmonary diseases in laboratory experiment [25]. Previous researches have indicated that Hla secreted by* S. aureus* is the main caused of cell injury in* S. aureus *and A549 cells coculture system [26, 27]. The potential protection of POG on Hla-mediated cell injury was verified in the* S. aureus* USA300 and A549 cells coculture system with different concentrations of POG. After being stained with the live/dead reagent, the live and dead cells showed green and red fluorescence, respectively. A549 cells without* S. aureus* showed green fluorescence in confocal laser scanning microscope (Figure 6(a)). In the drug-free coculture system, the rate of cell death significantly increased compared with A540 cells (without* S. aureus*). Under the confocal laser scanning microscope, more red fluorescent cells appeared in the drug-free coculture system (Figure 6(f)), also in the lowest drug concentration (Figure 6(e)). However, the amount of red fluorescence was significantly decreased in the coculture system treated with 8 *μ*g/mL of POG (Figure 6(d)) and treated with 16 *μ*g/mL of POG (Figure 6(c)). POG showed protection for A549 cells in 32 *μ*g/mL, as shown by almost no notice of red fluorescence (Figure 6(b)).

**Figure 7:**
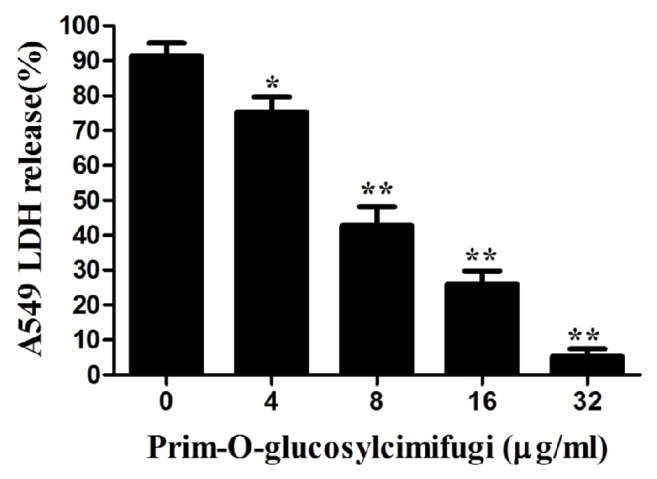
LDH release by A549 cells was quantified using a microplate reader at 490 nm. The samples were tested after exposure to certain concentrations of prim-O-glucosylcimifugin. The data are displayed as the means from three independent experiments. *∗*P < 0.05 and *∗∗*P < 0.01, compared with prim-O-glucosylcimifugin-free culture.

When the cell walls are destroyed by Hla, lactate dehydrogenase (LDH) will release to the culture medium. There is proportionality between the content of LDH in culture medium and the degree of cells damage. The concentration of LDH in coculture mediums was quantitatively estimated by LDH release assay kit. The amounts of LDH in the mediums showed the effect of POG to protect the influence of* S. aureus* USA300 on A549 cells. The results are presented as percentages of cell death. A dose-dependent reduction was shown at the concentrations ranging from 4 to 32 *μ*g/ml of POG in the coculture systems (Figure 7). The level of LDH release was 94.8% in the coculture system without POG. However, the level of cell death was decreased to 5.3% in the system treated with 32 *μ*g/mL of POG. Previous experiments demonstrated that POG is not impacting the growth of* S. aureus*. Consequently, it can be concluded that POG protected A549 cells by inhibiting the Hla production and decreasing* S. aureus* CFUs.

## 4. Discussion

The discovery of antibiotics and other antimicrobial therapies is one of major medical advances in modern medicine. Antibiotics have saved a great many people, particularly in bacterial infections. However, the extensive and long-term antibiotics use was leading to a remarkable increase in antibiotic resistance among bacteria, as antibiotics are increasing the survival selective pressure on the growth of bacteria. In clinic, more antibiotics or higher doses are used to treat drug-resistant bacterial infections. This situation were more costly in treatment or more toxic for patients [28, 29]. With the increasing resistance and the lack of development of new antibiotics, drug-resistant bacterial infections have become a major global health trouble. New drugs, especially drugs for new targets and new mechanisms are in urgent need of resistance bacterial infections. In this study, we found that POG can inhibit* S. aureus* reproduction under the high concentration (MIC=128 *μ*g/mL) and reduced the production of Hla at the low concentration. POG could be an ideal lead compound for anti-*S. aureus* infections.

MRSA is a familiar type of bacteria that is resistant to several antibiotics. The MRSA was identified as one of six different to treat ESKAPE pathogens (*Enterococcus, Staphylococcus, Klebsiella, Acinetobacter, Pseudomonas, *and* Enterobacter*) by the Antimicrobial Availability Task Force of the Infectious Diseases Society of America [30]. In 1961, after the application of methicillin (1960), MRSA isolates were first reported by British scientists [31]. Soon afterwards, MRSA was widespread worldwide [32, 33]. The CDC reported that two percent of people carry MRSA. In clinic, MRSA infections are treated with antibiotics. With a range of antibiotics application, MRSA is resistant to many common practice antibiotics. That means MRSA infections can be more difficult to treat than nonresistance bacteria. According to WHO, it is considered that the mortality probability of people infected with MRSA strains is higher than that of people infected with nonresistant* S. aureus* strains [34]. The data from CDC showed that more than 94,000 people were infected with MRSA, and the number of MRSA-related deaths is at least 19,000 people each year in USA [7]. MRSA remains an important pathogen that cause of hospital-acquired and community-associated infections worldwide. In the hospital and community, people were infected with MRSA, which led to severe diseases, such as pneumonia, bacteremia, and surgical site infections. USA300, a common community-associated MRSA strain, causes severe infection in healthy population worldwide [35–38]. CDC reported that 64% of MRSA isolates were USA300 strain in infected patients in 2006. USA300 strain, a really big issue in public health, can cause life-threatening illness.

The pathogenicity of* S. aureus* infection depends on the virulence factors that contain surface proteins, extracellular toxins, and enzymes produced from* S. aureus*.* S. aureus* excreted these virulence factors to assist bacteria adhesion and destruction to host cells, avoidance of the host immune defense, growth, and spread in host. Alpha-hemolysin, which is an important virulence protein, secreted by* S. aureus *into the extracellular, causes diseases by damaging various cells and tissues. Pneumocytes are effective target cells of Hla. Hla plays an important role in the pathogenesis of* S. aureus* pneumonia. Bubrck Wardenburg et al. (2007) have demonstrated that mice infected with* S. aureus* Hla mutation strains that were lacking in the Hla gene showed significantly less lung injury than mice infected with wild-type strains [14]. Hla is proved to be an ideal target for the drug development of therapeutics in many bacteria that secrete hemolysin [39–41]. Many studies had showed that some small molecules can control the staphylococcal aureus pneumonia by inhibiting the expression and activation of Hla in animal disease models [39, 42]. Hla is proved a virulence factor in* S. aureus* USA300 [43]. In our research, we found that the production of Hla was influenced by the concentration of prim-O-glucosylcimifugin. Hla productions are regulated by the accessory gene regulator (agr) quorum-sensing system in* S. aureus*.* RNAIII*, which is the major transcript of the* agr* operon, directly affects the expression of Hla [44]. The expression of* RNAIII* was also investigated in this study.

Many studies have proved that the production of Hla was increased, when* S. aureus* was in low concentrations of antibiotics (*β*-lactams and fluoroquinolones) [45, 46].* S. aureus* can strengthen Gram-negative bacterial pathogenicity by its Hla in mice pneumonia model [47]. Our results have proved that POG reduced the expression of Hla. POG may have the potential for treatment mixed-infection (G- +* S. aureus*) combination with common antibiotics, especially in* S. aureus* excreted Hla.

## 5. Conclusion

In this study, prim-O-glucosylcimifugin reduced Hla secretion without affecting the growth of* S. aureus* and showed the dose-dependent manner. In the cell experiments, A549 cells were protected from Hla injured by POG. POG could be developed as an antivirulence drug used in diseases caused by Hla.

## Figures and Tables

**Table 1:** Primers used in real-time RT-PCT.

Primer	Sequence	Location within gene
16S rRNA-forward	5′-GCTGCCCTTTGTATTGTC-3′	287-305
16S rRNA-reverse	5′-AGATGTTGGGTTAAGTCCC-3′	446-465
*hla*-forward	5′-TTGGTGCAAATGTTTC-3′	485-501
*hla*-reverse	5′-TCACTTTCCAGCCTACT-3′	569-586
*RNAIII*-forward	5′-TTCACTGTGTCGATAATCCA-3′	367-386
*RNAIII*-reverse	5′-GGAAGGAGTGATTTCAATGG-3′	428-447

## Data Availability

All relevant data are included within the paper. For further details, please contact yinlizi@hotmail.com.
